# A Review on Applications of Computational Methods in Drug Screening and Design

**DOI:** 10.3390/molecules25061375

**Published:** 2020-03-18

**Authors:** Xiaoqian Lin, Xiu Li, Xubo Lin

**Affiliations:** 1 Institute of Single Cell Engineering, Beijing Advanced Innovation Center for Biomedical Engineering, Beihang University, Beijing 100191, China; parkie@buaa.edu.cn; 2 School of Biological Science and Medical Engineering, Beihang University, Beijing 100191, China; 3 School of Chemistry and Material Science, Shanxi Normal University, Linfen 041004, China; zsypumc2008@163.com

**Keywords:** multiscale models, virtual screening, de novo design, machine learning

## Abstract

Drug development is one of the most significant processes in the pharmaceutical industry. Various computational methods have dramatically reduced the time and cost of drug discovery. In this review, we firstly discussed roles of multiscale biomolecular simulations in identifying drug binding sites on the target macromolecule and elucidating drug action mechanisms. Then, virtual screening methods (e.g., molecular docking, pharmacophore modeling, and QSAR) as well as structure- and ligand-based classical/de novo drug design were introduced and discussed. Last, we explored the development of machine learning methods and their applications in aforementioned computational methods to speed up the drug discovery process. Also, several application examples of combining various methods was discussed. A combination of different methods to jointly solve the tough problem at different scales and dimensions will be an inevitable trend in drug screening and design.

## 1. Introduction

With the rapid development of both computer hardware, software, and algorithms, drug screening and design have benefited much from various computational methods which greatly reduce the time and cost of drug development. In general, bioinformatics can help reveal the key genes from a massive amount of genomic data [1,2] and thus provide possible target proteins for drug screening and design. As a supplement to experiments, protein structure prediction methods can provide protein structures with reasonable precision [3]. Biomolecular simulations with multiscale models allow for investigations of both structural and thermodynamic features of target proteins on different levels [4], which are useful for identifying drug binding sites and elucidating drug action mechanisms. Virtual screening then searches chemical libraries to provide possible drug candidates based on drug binding sites on target proteins [5,6,7]. With greatly reduced amount of possible drug candidates, in-vitro cell experiments can further evaluate the efficacy of these molecules. In addition to virtual screening, de novo drug design methods [8], which generate synthesizable small molecules with high binding affinity, provide another type of computer-aided drug design direction. Artificial intelligence, e.g., machine learning and deep learning, is playing more and more important roles in the aforementioned computational methods and thus drug development [9,10,11]. In this review, we will focus on developments of the last four computational methods as well as their applications in drug screening and design.

## 2. Biomolecular Simulations in Drug Screening and Design

The Nobel Prize in Chemistry 2013 was awarded jointly to Martin Karplus, Michael Levitt, and Arieh Warshel for their pioneering contributions in the development of multiscale models for complex biochemical systems, recognizing the important role that theory and computational methods play as a direct and necessary complement to experiments [12]. Further, applications of biomolecular simulations in identifying drug binding sites and elucidating the molecular mechanisms of diseases have been subject to rapid developments [13,14,15].

Depending on the biological problems to be studied, multiscale models will be used in biomolecular simulations. The combination of quantum mechanics (QM) and molecular mechanics (MM) (QM/MM) can be used to study the electronic properties [16], simulate chemical reactions (e.g., the enzyme catalysis mechanism [17]), and calculate spectra [18] in a single simulation, which can be used to elucidate the action mechanism of certain drugs. Another widely used biomolecular method is molecular dynamics (MD) simulation [19,20,21,22,23,24], which applies empirical molecular mechanics (MM) force fields and is based on classical Newtonian mechanics. According to different accuracy requirements, all-atom (AA), united-atom (UA), and coarse-grained (CG) MD simulations as well as explicit/implicit solvent models, which allow simulations of temporal and spatial scales, can be used to facilitate the drug discovery [25,26]. Generally, MD simulations have been used for the identification of potential drug binding sites on target proteins, the calculation of binding free energy between target proteins and drug molecules, the action mechanism of drug molecules, etc. [27,28]. However, it is worth mentioning that MD simulations are also limited to time and length scales. Currently, AAMD simulations can explore time-dependent phenomena of large systems such as viral capsids in atomic detail as long as microseconds or even milliseconds [21,29,30]. Therefore, different types of simulation methods are required for different types of problems. Each of the different simulation methods has advantages, disadvantages, and practical limitations in terms of the size of system that can be simulated, the length of simulation that can be achieved, and the types of phenomena that can be modeled. On the other hand, improved algorithms, fast-growing data sets and computing ability are driving rapid advances in multiscale modeling methods which provide a powerful emerging paradigm for drug discovery.

As mentioned above, multiscale biomolecular simulations are useful in drug design. For example, MD simulations can be used to identify the drug binding sites on the target protein as several simple tools (e.g., fpocket [31]). Moreover, multiscale biomolecular simulations can be used to reveal the action mechanism of a drug, which happens mainly on a molecular level, but clearly has macroscopic effects [32,33,34]. From the molecular structure level to cellular tissue, the dynamics of drug targets and the surprising complexity of biological systems challenge our scientific understanding. In order to reveal how changes at different levels are linked and interaction network, no single simulation method can solve all these problems involved. Multiscale simulation methods are designed to simulate and analyze cross-scale connections, for example, how one scale change leads to another scale change. An obvious challenge is the integration of data and simulation across length scales and time scales. The current multiscale approach has potential to overcome these limitations by directly combining different levels of descriptions, thus bringing new prospects for drug discovery.

Multiscale simulations play an important role in studying biological processes. Here, we show applications of a combination of different methods to deal with complicated biological processes in this field. In recent years, although MD simulations are widely used, they still cannot consider the change of ionization states in simulated process. For example, MD simulations cannot discuss time dependence in a long time-scale of biological proton transport process, so the time dependence of protein changes with pH also cannot be revealed [35]. Therefore, in calculations and simulation process, it is necessary to combine the Monte Carlo (MC) approach (performed time-dependent simulations) in the proton transport process with the MD approach (performed pH dependent) in a protein model [35]. According to the electrostatic energy of the CG model, which can obtain the free energy of protonation states. In addition, the movement of the MC approach is based on the electrostatic energy of the CG model, and then proton transport time is used to make the scale, so as to correspond to the rate constant predicted by the transition state theory. Hence, they used isomorphism between probabilities obtained from the MC process and probability factors obtained from transition state theory [36], and converted the MC process to a time-dependent simulation with additional simplified modifications [37].

## 3. Drug Design and Virtual Screening

The design, discovery, and development of drugs are complex processes involving many different fields of knowledge and are considered a time-consuming and laborious inter-disciplinary work [38,39,40,41]. Different drug design methods and virtual screening will be very useful to design and find rational drug molecules based on the target macromolecule that interacts with the drug and thus speed up the whole drug discovery process. Here, we will discuss structure-based drug design, ligand-based drug design, and virtual screening.

### 3.1. Structure-Based Drug Design

Structure-based drug design must be performed with available structural models of the target proteins, which are provided by X-ray diffraction, nuclear magnetic resonance (NMR) or molecular simulation (homologous protein modeling, etc.) [42,43,44,45,46]. Keeping in mind the complexity of cancers which show diverse phenotypes and multiple etiologies, a one-size-fits-all drug design strategy for the development of cancer chemotherapeutics does not yield successful results. Lately, Arjmand et al. [47] adopted a series of methods, such as the combination of X-ray crystal structures and molecular docking, to design, synthesize, and characterize novel chromone based-copper(II) antitumor inhibitors. In general, after obtaining the structure of the receptor macromolecule by x-crystal single-crystal diffraction technique or multi-dimensional NMR, molecular modeling software can be used to analyze the physicochemical properties of drug binding sites on the receptor, especially including electrostatic field, hydrophobic field, hydrogen bond, and key residues. Then, the small molecule database is searched, or the drug design technique is used to identify the suitable molecules whose molecular shapes match the binding sites of the receptor and binding affinity is high. Then, these molecules are synthesized and their biological activities will be tested for further drug development. In short, structure-based drug design plays an extremely important role in drug design.

### 3.2. Ligand-Based Drug Design

Unlike structure-based drug design, ligand-based drug design doesn’t search small molecule libraries. Instead, it relies on knowledge of known molecules binding to the target macromolecule of interest. Using these known molecules, a pharmacophore model that defines the minimum necessary structural characteristics a molecule must possess in order to bind to the target can be derived [48,49]. Then, this model can be further used to design new molecular entities that interact with the target. On the other hand, ligand-based drug design can also use quantitative structure–activity relationships (QSAR) [50,51] in which a correlation between calculated properties of molecules and their experimentally determined biological activity is derived, to predict the activity of new analogs. Both the pharmacophore model and QSAR model will be discussed in detail in the following sessions.

### 3.3. Virtual Screening

In recent years, the rapid development of computational resources and small molecule databases have led to major breakthroughs in the development of lead compounds. As the number of new drug targets increases exponentially, computational methods are increasingly being used to accelerate the drug discovery process. This has led to the increased use of computer-assisted drug design and chemical bioinformatics techniques such as high-throughput docking, homology search and pharmacophore search in databases for virtual screening (VS) technology [51]. Virtual screening is an important part of computer-aided drug design methods. It may be the cheapest way to identify potential lead compounds, and many successful cases have proven successful using this technology.

The primary technique for identifying new lead compounds in drug discovery is to physically screen large chemical libraries for biological targets. In experiments, high-throughput screening identifies active molecules by performing separate biochemical analysis of more than one million compounds. However, this technology involves significant costs and time. Therefore, a cheaper and more efficient calculation method came into being, namely, virtual high-throughput screening. The method has been widely used in the early development of new drug. The main purpose is to determine the novel active small molecule structure from the large compound libraries. It is consistent with the purpose of high-throughput screening. The difference is that virtual screening can save a lot of experimental costs by significantly reducing the number of compounds for the measurement of the pharmacological activity, while high-throughput screening needs to perform experiments with all compounds in the database. Here, we will discuss common methods of virtual screening.

#### 3.3.1. Molecular Docking

Molecular docking, which predicts interaction patterns between proteins and small molecules as wel as proteins and proteins, to evaluate the binding between two molecules [52], is widely used in the field of drug screening and design. The theoretical basis is that the process of ligand and receptor recognition relies on spatial shape matching and energy matching, which is the theory of “inducing fit”. Determining the correct binding conformation of small molecule ligands and protein receptors in the formation of complex structures is the basis for drug design and studying its action mechanism. Molecular docking can be roughly divided into rigid docking, semi-flexible docking and flexible docking. In rigid docking, the structure of molecules does not change. The calculation method is relatively simple, and mainly studies the degree of conformation matching, so it is more suitable for studying macromolecular systems, such as protein–protein, protein–nucleic acid systems. In semi-flexible docking, the conformation of molecules can be varied within a certain range, so it is more suitable to deal with the interaction between proteins and small molecules [53]. In general, the structure of small molecules can be freely changed, while macromolecules remain rigid or retain some of the rotatable amino acid residues to ensure computational efficiency. In flexible docking, the simulated system conformation is free to change, thus consuming more computing resources while improving accuracy. What’s more, the establishment of binding sites in molecular docking methods is very important. For the first time, Collins [54] successfully determined the binding sites on the surface of proteins using a multi-scale algorithm and performed flexible docking of molecules, which greatly promoted the development of molecular docking.

#### 3.3.2. Pharmacophore Modeling

A pharmacophore is an abstract description of molecular features necessary for molecular recognition of a ligand by a biological macromolecule, which explains how structurally diverse ligands can bind to a common receptor site. When a drug molecule interacts with a target macromolecule, it produces a geometrically and energetically matched active conformation with the target. Medicinal chemists found that different chemical groups in drug molecules have different effects on activity, and changes to some groups have a great influence on the interaction between drugs and targets, while others have little effect [55]. Moreover, It was found that molecules with the same activity tend to have some of the same characteristics. Therefore, in 1909, Ehrlich proposed the concept of pharmacophores, which referred to the molecular framework of atoms with active essential characteristics [56]. In 1977, Gund further clarified the concept of pharmacophores as a group of molecules that recognize receptors and form structural features of molecular biological activity [57].

There are two main methods for the identification of pharmacophores. On one hand, if the target structure is available, the possible pharmacophore structure can be inferred by analyzing the action mode of receptor and drug molecule. On the other hand, when the structure of the target is unknown or the action mechanism is still unclear, a series of compounds will be studied for pharmacophores, and information on some groups that play a key role in the activity of compound will be summarized by means of conformational analysis and molecular folding [58]. Active compound that is suitable for constructing the model will be selected in the pharmacophore recognition process. Then, conformation analysis is used to find the binding conformation of molecule, and to determine the pharmacophore [59]. In recent years, with the development of compound databases and computer technology, the virtual screening of databases using the pharmacophore model has been widely used, and has become one of the important means to discover lead compounds.

#### 3.3.3. Quantitative Structure–Activity Relationship (QSAR)

QSAR is a quantitative study of the interactions between small organic molecules and biological macromolecules. It contains a correlation between calculated properties of molecules (e.g., absorption, distribution, metabolism of small organic molecules in living organisms) and their experimentally determined biological activity [51]. In the case of unknown receptor structure, the QSAR method is the most accurate and effective method for drug design. Drug discovery often involves the use of QSAR to identify chemical structures that could have good inhibitory effects on specific targets and have low toxicity (non-specific activity). With the further development of structure–activity relationship theory and statistical methods, in the 1980s, 3D structural information was introduced into the QSAR method, namely 3D-QSAR. Since 1990s, with the improvement of computing power and the accurate determination of 3D structure of many biomacromolecules, structure-based drug design has gradually replaced the dominant position of quantitative structure-activity relationship in the field of drug design, but QSAR with the advantages of small amount of calculation and good predictive ability [60] still plays an important role in pharmaceutical researches.

Based on 3D structural characteristics of ligands and targets, 3D-QSAR explores the 3D conception of bioactive molecules, accurately reflects the energy changes and patterns of interactions between bioactive molecules and receptors, and reveals the drug-receiving mechanism of body interactions. The physicochemical parameters and 3D structural parameters of a series of drugs are fitted to the quantitative relationship. Then, the structures of new compounds are predicted and optimized. In short, 3D-QSAR is actually a research method combining QSAR with computational chemistry and molecular graphics. It is a powerful tool for studying the interactions between drugs and target macromolecules, speculating the image of simulated targets, establishing the relationship of drug structure activity, and designing drugs.

## 4. Multiscale De Novo Drug Design toward Personalized Medicine

Computer-based de novo design methods of drug-like molecules are mainly for generating small molecule compounds with ideal physicochemical and pharmacological properties. In the past decades, fragment-based drug discovery had appeared as a novel concept that has proved a good prospect for improving lead optimization, in order to decrease the clinical attrition rates in drug design. It is an approach that uses small molecular fragments to deduce the biomolecular targets [61]. Fragment-based de novo design has obtained the long-term clinical success [62].

Despite the fact that modern drug discovery has made some successes in offering effective drugs, drug design has been affected by several factors, such as the tremendous chemical space for exploring drug molecules [63]. Further, as a large number of data increase in biological, chemical, and clinical medicine, it is obvious that the drug design should be solved with multiscale optimization methods, and concentrate on the data beyond molecular levels [64]. Thus, it is essential to discuss the function of multiscale models in drug discovery, and how they have predicted multiple biological properties in different biological targets. Accordingly, we discuss the combined application of both the concept of fragment-based on de novo design and multiscale modeling.

### 4.1. De Novo Drug Design Method

The fragment-based de novo design method starts with small building blocks. The initial molecular building blocks with desired properties are either elaborated upon (growing), directly connected (joining), or connected by a linker (linking). This process can be iterated until one or more molecules with the desired properties are obtained. There are two methods, namely structure-based and ligand-based methods [65]. Structure-based de novo design method searches novel ligands by using the 3D structural information of the protein target, which are usually constructed directly in the target protein binding site and evaluated by calculating the interaction energy of the target protein with ligands. Nevertheless, in de novo ligand design method, the molecule structure of protein target is unknown, and the new molecule is suggested based structure analogous to the known ligand molecule.

### 4.2. Multiscale De Novo Drug Design: Quantum Chemical Approaches to Structure-Based and Ligand-Based QSAR Models

Nowadays, proteochemometrics has emerged as a relatively new discipline for drug discovery. In this filed, QSAR analysis is a powerful tool for the efficient virtual screening, which shows physicochemical properties of various compounds. Compared to the classical QSAR, the QM calculations use reactivity descriptors in ligand-based QSARs, which provides an implicit model and calculate an exact enthalpy contributions of protein-ligand interactions. However, for the ab initio fragment, molecular orbital calculation in the structure-based QSARs, which obtains an explicit model and a clear enthalpy, changes the binding energy in different additional conditions. Moreover, it also calculates the free energy contribution of ligand-target complexes formation in structure-based and ligand-based QSAR models. Using a large number of ligand-target complexes to discuss the change of their binding affinity, more accurate optimization steps can be conducted based on good prediction and interpretation models [66]. The key of any QSAR model is how to accurately describe the molecule, and QM approaches provides a better understanding to the molecular and structural characteristics of ligands and drugs, so as to solve the problems existed in drug discovery.

The QSAR models of different scales are built according to the different computational precision, multiscale-QSAR research object mainly refers to the structure description of the training set, and involves small molecules and macromolecules [67,68]. In micro-, meso- and macroscopic scales, different molecular approaches will be used. QM approaches are often used to perform precise calculations at microscales, such as atom-based QSAR. Molecular force field focuses on mesoscopic-scale simulation, such as fragment-based QSAR. And coarse-grained study mainly performed in the macroscopic scales, such as macromolecule-based QSAR and cell-based QSAR. Moreover, multi-scale can also be reflected by different dimensions used in different QSAR models. CoMFA is a technique of 3D fragment-based QSAR, which can complete skeleton transition and R-groups substitution, providing different structures for new drug design. Besides, derived from proteochemometrics (PCM) [69], the 2.5D kinochemometrics (KCM) approach using 3D descriptor for protein kinases and 2D fingerprints for ligands can greatly increase the efficiency as well as the precision compared the traditional 3D QSAR methods [70]. The multiscale QSAR provides effective predictions for drug design, which integrates QSAR more systematically and applies all existing QSAR methods effectively.

Multiscale de novo drug design is a novel concept that combines QSAR models, QM calculations [71] and fragment-based drug discovery (FBDD) [72]. Here, the importance of explicit molecular descriptors is shown in a model from a molecular structural point of view through QM calculations. With the assembly of reasonable molecular fragments, the objective of drug design method is to produce a certain novel molecule that display highest biological activity, absorption, metabolism, elimination (ADME) and lowest toxicity properties at different environments, which belong to the application range of QSAR models. The multiscale de novo drug design methods can efficiently handle a large amount of biochemical/clinical data and obtain the chemical characteristics in order to improve the properties of the drug molecule. It is considered to be a more effective and safer method to discover new therapeutic agents.

## 5. Machine Learning Methods Accelerate Drug Development

In the process of drug discovery, machine intelligence methods have mostly been used in the above-mentioned computational methods over the past few decades [73]. With the booming era of “big” data, machine learning methods have developed into deep learning approaches, which are a more efficient way for drug designers to deal with important biological properties from large amount of compound databases. Here, we introduce applications of machine learning methods in QSAR analysis as well as the recent advances in deep learning methods.

### 5.1. Classical QSAR methods

Decision trees (DTs) are a simple, interpretable and predictive machine learning method. Ordinarily, there are two fundamental steps, that is, selecting properties and pruning for the decision trees building. The selected properties are considered as internal nodes, the branch representing the test result on the molecule, and the leaf node as a classification label. In order to avoid the complexity of the decision tree, the pruning program is used to prune the established tree. The DT is a typical classification algorithm, which is widely used in the prediction and auxiliary diagnosis of the disease, such as management decision-making, the classification mode for creating the metabolic disorder, and data mining of diabetes etc. [74]. Abdul et al. [75] developed a task-based chemical toxicity prediction framework, and used a decision tree to obtain an optimum number of features from a collection of thousands of them, which effectively help chemists perform prescreening of toxic compounds effectively.

The artificial neural network (ANN) achieves problem-solving by mimicking brain function. Just as the brain applies information obtained from past experience to solve new problems, a neural network can construct a system of “neurons” that reaches new decisions, classifications, and prediction based on previous experiences. The processing element is similar to a neuron, and a massive processing element is organized by the layers. They include three types: input, hidden, and output layers. ANN benefits from high self-organization, robustness, and fault tolerance, and has been widely applied in prognosis evaluation and early prevention of diseases. Lorenzo et al. [76] used the interpretable ANN to predict biophysical properties of therapeutic monoclonal antibodies, include melting temperature, aggregation onset temperature and interaction parameters as a function of pH and salt concentration from the amino acid composition. Artificial neural networks had their first heyday in molecular informatics and drug discovery approximately two decades ago. Currently, we are witnessing renewed interest in adapting advanced neural network architectures for pharmaceutical research by borrowing ideas from the deep learning field. Compared with some other fields in life sciences, their applications in drug discovery is still limited.

The support vector machine (SVM) is one of the most promising machine learning methods that can use molecular descriptors to construct a predictive QSAR models and deal with high-dimensional datasets. ANN and multiple linear regression analysis were used to construct linear and nonlinear models, which were then compared with the results gained by SVM. For linear models, the SVM approaches use space mapping points to separate different classification for maximizing the range between different categories of points [77]. Further, for the nonlinear models, SVMs use nucleus mapping to transform into a high-dimension space for linear classification. At present, the SVM approach has been widely used in modeling at different scales for drug discovery [78].

The k nearest neighbor (kNN) is one of the simplest and most intuitive algorithms among all machine learning methods, and is usually jointly used with other selection algorithms in the feature space. Further, it is used for classification and regression based on example learning. Normally, molecules are classified by votes of its closest neighbors, resulting in the most common class that molecules are distributed to its closest neighbors. Here, the value of k is the number of closest neighbors. Based on ligand-based virtual screening, kNN can be viewed as a prolongation form chemical similarity searching to supervised learning, and the top search results predicted the best bioactivities. Weber et al. [79] tried two machine learning algorithms of classification (KNN and RF) to analyze genotype-phenotype datasets of HIV protease and reverse transcriptase (RT). As a result, both algorithms had high accuracies for predicting the drug resistance for protease and RT inhibitors.

The random forest (RF) is an ensemble learning approach involving the building of multiple DTs based on the training examples. Similar to kNN algorithms, it is also used to for classification and to predict regression [80]. Compared to DTs, it is impossible that RF over-fits the data, and the RF has been used for bioactivity data classification [81], toxicity modeling [82], and drug target prediction [83], etc. Wang et al. [84] used the RF approach to model the binding affinity of protein-ligand on 170 HIV-1 proteases complexes, 110 trypsin complexes, and 126 carbonic anhydrase complexes, which demonstrated that individual representation and model construction for each protein family is a more reasonable way in predicting the affinity of one particular protein family.

Currently, the multiscale models can predict toxicity, activity, and ADME properties of different proteins and microbial targets by integrating different genomes and proteomics. Cheminformatics has played an important role in rationalize drug discovery. The QSAR model has become the main auxiliary tool which can achieve virtual screening of various pharmacological characteristics. Although the QSAR model has been widely used in the search and design of new drug, classical QSAR models can only predict the activity and toxicity of one biomolecule against one certain target. However, the multi-target QSAR (mt-QSAR) can be used to carry out rational drug design at multiple targets, which provides a better way to understand various pharmacological characteristic molecules including antibacterial activity and toxicity. Furthermore, uniform multitasking models based on quantitative structure biological effect relationship (mtk-QSBER) have been used in a lot of researches. These models were built by ANN and the topological indices, which can predict the biological activity and toxicity correctly and classify the compounds in experimental conditions. Meanwhile, these models used perturbation models to form structural-activity relationships between the site of infection and the drug, such as the PTML model [85] and the ChEMBL model [86], which has been applied in infectious diseases [71], immunology [85], and cancer [87] widely. Currently, the mtk-QSBER model has been able to carry out the in-silico design and virtual screening of an antibacterial drug efficiently, and these antibacterial drugs have good biosafety. These methods have provided a powerful tool for in silico screening reasonable drugs.

### 5.2. Advances in Deep Learning Approaches

The deep learning network is a concept closely related to ANN, which are learning of the concept of layering. In other words, it is a multiple learning approaches ranging from low to high levels. Just when the molecular descriptors are not selected, the deep learning method will automatically select representations from original data and high-dimensional data [88]. Therefore, it allows deep learning to be applied to the model building of drug discovery [89]. The convolutional neural networks (CNN) are most commonly used, which have made great progresses in the computer vision community [90], and been applied in the drug design fields including de novo drug molecule identification, protein engineering and gene expression analysis. With the rapid development of deep learning concepts such as CNN, the molecular modeler’s tool box has been equipped with potentially game-changing methods. Judging from the success of recent pioneering studies, we are convinced that modern deep learning architecture will be useful for the coming age of big data analysis in pharmaceutical research, toxicity prediction, genome mining and chemogenomic applications, to name only some of the immediate areas of application. Kiminori et al. [91] developed a fundamental technology that can predict the resistance of free cancer cells to fluorinated pyrimidine anticancer drugs by deep learning from the morphological image data taken from images. Cai et al. [92] developed a deep learning approach, termed deep human ether-a-go-go-related gene (hERG), for the prediction of hERG blockers of small molecules in drug discovery and post-marketing surveillance. The group found that deephERG models built by a multitask deep neural network (DNN) algorithm outperformed those built by single-task DNN, SVM and RF.

Now, the drug development technologies usually include artificial intelligence-based (AI-based) techniques. Most AI applications only concentrate on limited tasks. Moreover, current AI can only direct patients’ specific problems, it cannot make subjective inferences like doctors with the overall physical context of a patient. As a subfield of AI, ML can be successfully used for training in the quality of examples. However, this process is very time-consuming and costly. The development of ML techniques and the application of existing algorithms to process massive amounts of digital data resulted in higher requirements for computer hardware, which also increases the clinical cost. DL, which is also a subset of ML, and can process big data and create patterns by layers of neurons. However, it is difficult to understand how each decision is obtained by algorithm. ML methods have achieved great successes in the field of chemoinformatics to design and discover new drugs. An important innovation is the combination of ML methods and big data analysis to predict more extensive biological features. It is vital to discover more secure and efficient drugs by integrating structural, genetic information and pharmacological data from the scale of molecular to organism [93]. In addition, DL approaches have proven to be a promising way for efficiently learning from a large variety of datasets for modern novel drug discovery.

## 6. Applications of Multiscale Methods in Drug Discovery

### 6.1. Molecular Dynamics of Cardiac Modelling

Multiscale modeling of the drugs in an excitable system is critical because experiments on a single system scale cannot reveal the underlying effects of multiple drug interactions. A computationally based approach to predict the emergency effects of drugs on excitatory rhythms may form an interactive technology-driven process for the drug and disease screening industry, research and development academia, and patient-oriented medical clinic. There are potentially far-reaching implications because millions of people affected by arrhythmia each year will benefit from improved risk stratification of drug-based interventions.

Much progress has been made in developing multiscale computational modeling and simulation approaches for predicting effects of cardiac ion channel blocking drugs. Structural modeling of ion channel interactions with drugs is a critical approach for current and future drug discovery efforts. Modeling of drug receptor sites within an ion channel structure can be useful to identify key drug-channel interaction sites. Drug interactions with cardiac ion channels have been modeled at the atomic scale in simulated docking and MD simulations, as well as at the level of channel function to simulate drug effects on channel behavior [32,94,95,96,97,98,99,100]. Structural modeling of drug-channel interactions at the atomic scale may ultimately allow for the design of novel high-affinity and subtype selective drugs for specific targeting of receptors for cardiac and neurological disorders.

### 6.2. Cancer Modeling and Network Biology

The World Health Organization (WHO) stated that cancer remains one of the most dangerous diseases today. Considering that cancer is a multifactorial disease, there is increasing interest in multi-target compounds that can target multiple intracellular pathways. However, the study of large data sets for the analysis of anticancer compounds is difficult, with a large amount of data and high data complexity. For example, the ChEMBL database [101] compiles big datasets of very heterogeneous preclinical assays. Bediaga et al. [87] have reported a PTML-LDA model of the ChEMBL dataset for the preclinical determination of anticancer compounds. PTML is a model that combines perturbation theory (PT) ideas and ML methods to solve similar problems. They compared this model with other PTML models which was reported by Speck-Planche et al. [72,102,103,104] and then concluded that this is the only one that can predict activity against multiple cancers. Speck-Planche et al. also derived a multi-task (mtk) chemical information model combining Broto Moreau autocorrelation with ANN from a dataset containing 1933 peptide cases. This model is used to virtually design and screen peptides with potential anti-cancer activity against different cancer cell lines and low cytotoxicity to a variety of healthy mammalian cells, and the model shows greater than 92% in both training and prediction (test)accuracy.

In addition, due to the inherent complexity of tumors, it is necessary to analyze their growth at different scales. It includes many phenomena that occur at various spatial scales from tissue to molecular length. The complexity of cancer development is manifested in at least three scales that can be distinguished and described by mathematical models, namely microscale, mesoscale, and macroscale. Wang et al. conducted a number of studies on how to use multiscale models for the identification and combination therapy of drug targets [105,106,107,108,109,110,111]. This method is based on quantification of relationship between intracellular epidermal growth factor receptor (EGFR) signaling kinetics, lung cancer extracellular epidermal growth factor (EGF) stimulation and multicellular growth. The multiscale modeling of tumors combined with systemic pharmacology will contribute to the development of practical smart drugs. It will produce a comprehensive system-level approach to determine the dynamics and effects of existing and new drugs in preclinical trials, model organisms and individual patients. In addition, mathematical and computational studies will provide a better way to understand many factors that influence the effects of drugs, thus helping to uncover better ways to therapeutically interfere with disease.

### 6.3. Multiscale Modeling for Drug Discovery in Brain Disease

Multiscale models can be also used to identify pathophysiological processes to allow disease staging. In many cases, like cancer, treatments vary depending on the stage of disease. The model can help determine prognosis, which is an important clinical determination that can help determine the right type of medication to be administered or discovered. Several models focus on the neuronal network levels, including Cutsuridis and Moustafa for Alzheimer’s disease [112], and Lytton for epilepsy [113]. ANN is a class of ML techniques that can be used for clinical analysis of big data including that related to drug testing, which is critical for drug discovery. In addition, Anastasio [114] introduced process algebra, a computer technology widely used to analyze complex computing systems, used here to calculate neurology. Sirci et al. [115] described how network (map) theory is used to identify similarities and differences between different pharmacological agents. In this type of study, each drug is a node, and the edges between drugs represent chemical and transcription-based interactions that characterize the drug.

In addition, Ferreira da Costa et al. [116] report the first PTML (PT + ML) study of a large number of ChEMBL datasets for preclinical determinations of compounds for dopamine pathway proteins. Molecular docking or ML models can be used to solve a specific protein, but these models cannot explain the large and complex large data sets of preclinical assays reported in public databases. PT models, on the other hand, allow us to predict the properties of a query compound or molecular system in an experimental analysis with multiple boundary conditions based on previously known reference cases. In their work, the best PTML model found in the training and external validation series has an accuracy of 70–91%. Hansch’s model is a classic method for solving quantitative structural binding relationships (QSBR) in pharmacology and medicinal chemistry. Abeijon et al. [117] developed a new PT-QSBR Hansch model based on PT and QSBR methods for a large number of drugs reported in ChEMBL, focusing on a protein expressed in the hippocampus of the brain of Alzheimer’s disease (AD) patients. Now, by decomposing how risks and causes are combined in complex systems to produce disease, and how to prevent or improve these diseases through multi-stage, multi-target, multi-drug techniques, multiscale modeling is gradually being grasped.

### 6.4. Infectious Diseases

From AIDS, hepatitis C, influenza, and other disease-related viruses to the current 2019-nCoV, we have been working hard to develop antiviral drugs targeting them. However, the unique structure and proliferation of the virus pose a natural challenge for drug development. Viruses do not have their own cellular structure and metabolic system, and must replicate and proliferate in host cells. Therefore, it is difficult to find compounds that target only viral targets without affecting the normal function of host cells. At present, the main way that some antiviral drugs work is to inhibit viral replication. However, many of the tools used for virus replication come from human cells, such as ribosomes, and the corresponding antiviral drugs will also bring great side effects to the human body. Therefore, the discovery of drugs requires the introduction of a multi-scale model to screen out drugs that can inhibit viral replication while reducing the damage to the human body.

So far, retroviral infections, such as HIV, are incurable diseases. ChEMBL manages big data capabilities through complex datasets, which make the information difficult to analyze because these datasets describe numerous features for predicting new drugs for retroviral infections. Without proper model, it is impossible to make full use of these features. Hence, Vásquez-Domínguez et al. [118] proposed a PTML model for the ChEMBL dataset, which can be efficiently used for preclinical experimental analysis of antiretroviral compounds. The PT operator is based on a multi-conditional moving average, which combines different functions and simplifies the management of all data. The PTML model they proposed was the first to consider multiple features combined with preclinical experimental antiretroviral tests. In order to simultaneously explore antibacterial activity against Gram-negative pathogens and in vitro safety related to absorption, distribution, metabolism, elimination, and toxicity (ADMET), the Speck-Planchee et al. [119] further proposed the first mtk-QSBER model. The accuracy of this model in both the training and prediction (test) sets is higher than 97%. They also have developed a chemoinformatic model for simultaneous prediction of anti-cocci activities and in vitro safety [71]. The best model displayed accuracies around 93% in both training and prediction (test) sets. Additionally, focusing on exploring anti-hepatitis C virus (HCV), the accuracy shown in the training and prediction (test) sets is higher than 95% using this model [120]. Cytotoxicity is one of the main concerns in the early development of peptide-based drugs. Kleandrova et al. [121] introduce the first multi-task processing (mtk) computational model focused on predicting both antibacterial activity and peptide cytotoxicity. Gonzalez-Diaz et al. [122] developed a model called LNN-ALMA to generate complex networks of the AIDS prevalence with respect to the preclinical activity of anti-HIV drugs.

Multiscale models are also imperfect and have their limitations. Models are expressions and simplifications of real life. No model can represent everything that can happen in the system. All models contain specific assumptions, and models vary widely in their comprehensiveness, quality, and utility. In other words, each model can only solve limited problems. Hence, we need to integrate different computational models and data in order to make full use of these models.

## 7. Conclusions

Computational methods have come to play significant roles in drug screening and design. Multiscale biomolecular simulations can help identify the drug binding sites on the target macromolecules and elucidate the drug action mechanisms. Virtual screening can efficiently search massive chemical databases for lead compounds. De novo drug design provides alternative powerful way to design drug molecules from scratch using building blocks summarized and abstracted in previous successful drug discovery. ML is revolutionizing most computational methods in drug screening and design, which may greatly improve the efficiency and precision for the big data era. As we frequently emphasize, different models or efficient algorithms (e.g., dimensionality reduction) need to be integrated properly to achieve the comprehensive study of biological processes at multiple scales as well as accurate and effective drug screening and design. The integrated computational methods will accelerate drug development and help identify effective therapies with novel action mechanisms that can ultimately be applied to a variety of complex biological systems.
